# Dietary Supplementation of Tannic Acid Promotes Performance of Beef Cattle via Alleviating Liver Lipid Peroxidation and Improving Glucose Metabolism and Rumen Fermentation

**DOI:** 10.3390/antiox12091774

**Published:** 2023-09-18

**Authors:** Tengfei He, Guang Yi, Jiangong Li, Zhenlong Wu, Yao Guo, Fang Sun, Jijun Liu, Chunjuan Tang, Shenfei Long, Zhaohui Chen

**Affiliations:** 1 College of Animal Science and Technology, China Agricultural University, Beijing 100193, China; hetengfei@cau.edu.cn (T.H.); sy20203040712@cau.edu.cn (G.Y.); jli153@cau.edu.cn (J.L.); wuzhenlong@cau.edu.cn (Z.W.); guoyaocau@163.com (Y.G.); liujijun@cau.edu.cn (J.L.); 2 State Key Laboratory of Animal Nutrition and Feeding, Beijing 100193, China; 3 Institute of Animal Huabandry, Hei Longjiang Academy of Agricultural Sciences, Harbin 150086, China; hljxmsf@163.com; 4 Silvateam S.p.a, 12080 Cuneo, Italy; mary@silvateam.com

**Keywords:** tannic acid, performance, lipid peroxidation, rumen fermentation, beef cattle

## Abstract

This study aimed to investigate the effects of dietary tannic acid (TAN) on the gas production, growth performance, antioxidant capacity, rumen microflora, and fermentation function of beef cattle through in vitro and in vivo experiments. TAN was evaluated at 0.15% (dry matter basis, DM) in the in vitro experiment and 0.20% (DM basis) in the animal feeding experiment. The in vitro results revealed that compared with control (CON, basal diet without TAN), the addition of TAN significantly increased the cumulative gas production and asymptotic gas production per 0.20 g dry matter substrate (*p* < 0.01), with a tendency to reduce methane concentration after 96 h of fermentation (*p* = 0.10). Furthermore, TAN supplementation significantly suppressed the relative abundance of *Methanosphaera* and *Methanobacteriaceae* in the fermentation fluid (LDA > 2.50, *p* < 0.05). The in vivo experiment showed that compared with CON, the dietary TAN significantly improved average daily gain (+0.15 kg/d), dressing percent (+1.30%), net meat percentage (+1.60%), and serum glucose concentration (+23.35%) of beef cattle (*p* < 0.05), while it also significantly reduced hepatic malondialdehyde contents by 25.69% (*p* = 0.02). Moreover, the TAN group showed significantly higher alpha diversity (*p* < 0.05) and increased relative abundance of *Ruminococcus* and *Saccharomonas* (LDA > 2.50, *p* < 0.05), while the relative abundance of *Prevotellaceae* in rumen microbial community was significantly decreased (*p* < 0.05) as compared to that of the CON group. In conclusion, the dietary supplementation of TAN could improve the growth and slaughter performance and health status of beef cattle, and these favorable effects might be attributed to its ability to alleviate liver lipid peroxidation, enhance glucose metabolism, and promote a balanced rumen microbiota for optimal fermentation.

## 1. Introduction

Tannic acid (TAN) is a natural polyphenolic compound found in plants, capable of forming stable complexes with dietary proteins through hydrogen bonding in the rumen’s optimal pH range of 6.0–7.0 [1,2]. This property allows TAN to protect vegetable proteins from degradation by rumen proteases. However, as tannin–protein complexes pass into the abomasum, the lower pH (2.5–3.5) dissociates these complexes, resulting in reduced protein degradation by rumen microbes [3]. As a result, a larger amount of protein could pass through the rumen undigested and be subsequently digested and absorbed in the small intestine, improving dietary protein utilization [2]. Additionally, TAN exhibited inhibitory effects on rumen microorganisms, including methane-producing bacteria, leading to reduced fiber digestibility and methane production [1,4,5]. A meta-analysis conducted by Jayanegara et al. [6] also concluded that increased TAN content in the diet correlated with decreased methane emissions. In vitro fermentation studies by Wisam et al. [7] demonstrated that TAN addition had no effect on rumen fermentation pH, NH_3_-N, acetic acid to propionic acid ratio, or total volatile fatty acids, but resulted in reduced methane production.

Due to its polyphenolic and flavonoid structure, TAN exhibits potential antioxidant activity [8], as demonstrated by chemical and cellular antioxidant experiments [9]. Anthocyanins, a type of TAN, have shown an oxygen radical absorbance capacity of approximately 4500 μmol Trolox equivalents/mmol (oxygen radical absorbance capacity), indicating that other types of TAN might possess similar effects [10]. Furthermore, a previous study indicated that TAN could dose-dependently restore superoxide dismutase activity in thioacetamide-treated rats [11]. Calis et al. [12] also reported that TAN reduced malondialdehyde level in rat brain tissue homogenates and increased SOD activity in blood hemolysis, thereby alleviating oxidative stress in rats subjected to sodium glutamate monohydrate treatment. These findings suggest that TAN might have the potential to serve as an antioxidant additive in ruminant diets [13].

Oxidative stress in beef cattle, mainly induced by factors such as diet composition [14,15], rearing environment [16], and transportation [17], might be experienced throughout their growth and development, which could highly impair the growth performance and meat quality of beef cattle [14]. Current research on TAN in ruminants mainly focuses on methane (CH_4_) emission [5] and nitrogen utilization [5,18]. However, there is still a lack of clear understanding regarding the effects of TAN on the antioxidant capacity and health of beef cattle. Therefore, the purpose of this study was conducted to comprehensively investigate the effects of TAN on in vitro gas production, antioxidant, immune, and anti-inflammatory capabilities, and rumen fermentation of beef cattle through in vitro and in vivo experiments. The aim is to gain insight into the role of TAN in promoting performance in beef cattle and provide a scientific basis for its application in the diet.

## 2. Materials and Methods

The experiments were approved by the Animal Care and Use Committee of China Agricultural University (approval number AW71012022-1-3) and were carried out at the Beef Cattle Research Station of China Agricultural University in Beijing. The product (Silvafeed Bypro) used in this study was supplied by Silvateam S.p.a, Italy, and its composition included chestnut tannin extract and quebracho tannin extract.

### 2.1. In Vitro Culture Procedure

The in vitro fermentation was conducted following the method of Menke et al. [19]. Fresh rumen fluid was collected 2 h before the morning feeding from 3 Angus cattle (460 ± 48 kg) with permanent rumen fistulas. The rumen fluid was then filtered through four layers of gauze and mixed with artificial saliva in a 1:2 volume ratio with CO_2_ continuously introduced to maintain an anaerobic environment. Substrates (Formulation as Table S1) with or without 0.15% TAN (dry matter basis, DM) weighing 220 mg were placed in 6 incubation tubes (D-89173, Haberle Labortechnik, Lonsee, Germany), with 3 replicates per treatment, 1 culture tube per replicate. A total of 30 mL of inoculum was injected into each tube using a Varispenser (Eppendorf AG, Hamburg, Germany). The incubation tubes were then quickly transferred into a water bath shaker (Jie Cheng Experimental Apparatus, Shanghai, China) maintained at 39 °C. The cumulative gas production was manually recorded at 0, 2, 4, 6, 8, 10, 12, 18, 24, 30, 36, 42, 48, 60, 72, 84, and 96 h during the incubation. Three gas samples were collected from each tube after incubating for 96 h, and the CH_4_ and CO_2_ production in each injection was determined by gas chromatography (TP-2060F, Beijing Beifen Tianpu Analytical Instrument Co., Ltd., Beijing, China). Fermentation fluid was sampled from each tube and then centrifuged at 8000× *g* for 15 min at 4 °C, and the supernatant was put into 2.0 mL sterilized cryopreservation tubes (NEST Biotechnology Co., Ltd., Wuxi, China) and quickly stored in liquid nitrogen for subsequent sequencing microbial composition analysis.

### 2.2. Animals, Design and Management

A total of 19 beef cattle (550 ± 27.5 kg) aged 19 to 20 months were randomly divided into 2 treatments, with 10 replicates in the control group (CON, basal diet) and 9 replicates in the TAN group (the basal diet supplemented with TAN at 0.20% DM), replicate with 1 cattle and raised individually. The period lasted for 65 d, conducted in the last 2 months of fattening of cattle until the slaughter, divided into the early stage (d 0–31) and the late stage (d 31–65). The basal diet for the experiment was shown in Table 1. The method of adding TAN to the diet was based on previous studies [1,13]. Briefly, TAN substituted for corn in the basal diet and was provided in powdered form. Initially, TAN was pre-mixed with cornmeal, then mixed using a feed mixer (Tiejia Agricultural Machinery Co., Ltd.; Dezhou, China), and the diets were fed twice a day, at 7:00 and 15:00, respectively, as the form of total mixed rations (TMR). The remaining feed from the previous day was removed at 6:30. During the experiment period, the beef cattle were ensured ad libitum feeding.

### 2.3. Sample Collecion

At the beginning, midpoint, and termination of the experiment, the individual weights of the cattle were measured for three consecutive mornings before feeding, from which the ADG was subsequently calculated. Cattle (*n* = 5) with body weights close to the mean in each group were selected for further study analysis of nutrient digestibility, blood, rumen fluid, and meat samples.

TMR and fresh fecal samples (300 g each) from each treatment group were collected over three consecutive days at the end of the experiment. These samples were amalgamated, and a 300 g subsample was taken for drying—the subsample was dried at 55 °C for 72 h and subsequently ground to a 2 mm size using a Wiley mill (Arthur H. Thomas Co., Philadelphia, PA, USA). This ground sample was then set aside in the refrigerator at 4 °C for further nutritional analysis.

Approximately 5 mL of blood was collected from the cattle via the tail vein using heparinized tubes before morning feeding on d 66. The collected blood samples were immediately centrifuged at 3000× *g* for 15 min at 4 °C to obtain serum and then stored in a −80 °C refrigerator. The samples of ruminal fluid were also collected 3 h after morning feeding on d 66 by aspiration using an esophagogastric tube. During rumen fluid extraction, the initial 200 mL was discarded. The remaining fluid was then filtered through four layers of sterile gauze. Subsequently, this filtered rumen fluid was aliquoted into three 2.0 mL sterile storage tubes (NEST Biotech Co. Ltd., Wuxi, China) and was cryopreserved in liquid nitrogen for future analysis. On the 67th day, the cattle were transported to a commercial slaughterhouse for humane slaughtering. Pertinent data including pre-slaughter weight, hot carcass weight, net meat weight, and bone weight were documented. Concurrently, samples were extracted from the longest dorsal muscle for meat quality assessment. Post-slaughter, liver tissue samples were promptly harvested from the median lobe, minced, and stored in 2.00 mL sterile storage tubes (manufacturer: Naisite Biotech Co. Ltd., Nanjing, China). These samples were then rapidly immersed in liquid nitrogen in preparation for subsequent sequencing analysis.

### 2.4. Chemical Analysis

#### 2.4.1. Nutrient Digestibility

Comprehensive evaluations were performed on the principal nutritional components within the TMR and fecal samples. By employing the methods stipulated by AOAC [20], the contents of dry matter (DM), crude ash (Ash), crude protein (CP), and ether extract (EE) were quantified. The content of organic matter (OM) was derived by subtracting the ash content from the total. The levels of neutral detergent fiber (NDF) and acid detergent fiber (ADF) were determined in accordance with the methodologies presented by Van Soest et al. [20]. For the assessment of nutrient digestibility, the acid-insoluble ash method introduced by VanKeulen and Young [21] was adopted. The formula for this measurement was:

D = [1 − (Ad × Nf)/(Af × Nd)] × 100

In this equation, Ad (g/kg) and Af (g/kg) denote the acid-insoluble ash content in the feed and feces, respectively, while Nd (g/kg) and Nf (g/kg) correspond to the nutrient contents in the feed and feces, respectively.

#### 2.4.2. Serum and Liver Biochemical Indicators

Serum and liver biochemical indicators, including total antioxidant capacity (T-AOC), superoxide dismutase (SOD), malondialdehyde (MDA), glucose (Glu), insulin (INS), total cholesterol (TC), triglyceride (TG), total protein (TP), albumin (ALB), and blood urea nitrogen (BUN) were analyzed using the commercial kits according to the instructions (Nanjing Jiancheng Bioengineering Research Institute, Nanjing, China) by a CLS880 fully automatic biochemical analyzer (Zecen Biotech, Taizhou, China). Serum and liver immunoglobulin A (IgA), immunoglobulin G (IgG), tumor necrosis factor-α (TNF-α), interleukin-10 (IL-10), heat shock protein 70 (HSP-70), and insulin-like growth factor-1 (IGF-1) were quantified using the method of enzyme-linked immunosorbent assay, which was performed following the instructions provided with the kit (Jiangsu Enzyme Industrial Co., Ltd., Taizhou, China).

#### 2.4.3. Rumen Fermentation Parameters

The concentration of ammonia nitrogen was determined following the method delineated by Broderick and Kang [22], and the subsequent quantification was carried out using a Shimadzu UV-1700 spectrophotometer (Shimadzu Corporation, Kyoto, Japan). The volatile fatty acids (VFA) were quantified utilizing a GC-8600 high-performance gas chromatograph (Beifen-Ruili Instrument Technology Co., Ltd., Beijing, China).

### 2.5. Meat Quality Analysis

The pH value was gauged 45 min post-slaughter using a Cyberscan pH310 pH meter (EUTECH, Singapore). Chromaticity values, including luminosity (L*), redness (a*), and yellowness (b*), were tested using a chromameter manufactured by Shanghai Precision Scientific Instrument Co., Shanghai, China. Each sample underwent triplicate measurements at a consistent site to ascertain an average value. Uniformly shaped samples of the longissimus dorsi were vacuum-sealed, minimizing meat–bag wall contact, and subsequently suspended in a refrigeration setting at 4 °C for 24 h. Drip loss was calculated based on the subsequent formula:

Drip Loss = [(Initial sample weight − Weight post 24 h)/Initial sample weight] × 100%

### 2.6. Rumen Microbiota Analysis 

DNA extraction and PCR amplification: Total DNA of bacteria and methanogens was extracted using the E.Z.N.A.^®^ Soil DNA Kit (Omega Bio-tek, Norcross, GA, USA) and its quality confirmed by 1% agarose gel electrophoresis. PCR amplification of the 16S rRNA gene was performed using primers 338F (5′-ACTCCTACGGGAGGCAGCAG-3′) and 806R (5′-GGACTACHVGGGTWTCTAAT-3′) or MLfF (5′-GGTGGTGTMGGATTCACACARTAYGCWACAGC-3′) and MLrR (5′-TTCATTGCRTAGTTWGGRTAGTT-3′) for bacteria and methanogens, respectively. The amplification procedure consisted of an initial denaturation at 95 °C for 3 min, followed by 27 cycles of denaturation at 95 °C for 30 s, annealing at 55 °C for 30 s, extension at 72 °C for 30 s, and a final extension at 72 °C for 10 min. The PCR reaction was carried out using an ABI GeneAmp^®^ 9700 thermal cycler.

Illumina Miseq sequencing and data process: PCR products from the same sample were pooled, purified, and quantified. Library construction was carried out using the NEXTFLEX Rapid DNA-Seq Kit, including adapter linking, screening for self-ligated fragments, PCR amplification, and magnetic bead recovery. Sequencing was performed on the Illumina Miseq PE300/NovaSeq PE250 platform (Shanghai Meiji Biomedical Technology Co., Ltd., Shanghai, China). Quality control and read merging were conducted using fastp [23] (https://github.com/OpenGene/fastp, accessed on 11 June 2023) and FLASH [24] (http://www.cbcb.umd.edu/software/flash, accessed on 15 June 2023) software. The sequences were clustered into operational taxonomic units (OTUs) based on a similarity threshold of 97% [25], and chimeric sequences were removed. The RDP classifier [26] (http://rdp.cme.msu.edu/, version 2.2, accessed on 20 June 2023) was employed for species classification annotation of each sequence, using the Silva 16S rRNA database (v138) with a comparison threshold set at 70%.

### 2.7. Statistical Analysis

Based on the formula proposed by Ørskov and McDonald [27], the kinetic parameters of cumulative gas production were determined. The equation utilized was: Y = X × (1 − e^−ct^). In this context, Y signifies the gas volume (mL) produced per 0.2 g DM substrate at time t; X denotes the asymptotic gas production from 0.2 g DM substrate (mL); and c represents the hourly rate of gas generation.

The data except microbiota were organized and analyzed using the SAS 9.4 (SAS Institute Inc., Cary, NC, USA) through unpaired Student’s *t*-test. Utilizing the Kruskal–Wallis rank sum test, the Linear Discriminant Analysis Effect Size (LEfSe) was employed to assess variations in microbial community abundance within fecal samples. A significant distinction in effect size is denoted by an LDA score (threshold ≥ 2.50). A *p* < 0.05 was considered indicative of significant differences between treatments. Differences of 0.05 ≤ *p* ≤ 0.10 were considered a tendency.

## 3. Results

### 3.1. Fermentation Parameters of In Vitro Experiment

The results of in vitro fermentation (Table 2) revealed that, in comparison to the CON, the cumulative gas production from 0.20 g DM substrate during the incubation periods of 24, 48, 72, and 96 h and the asymptotic gas production in the TAN group was significantly elevated (*p* < 0.05). However, the hourly gas production rate exhibited a decline in the TAN group (*p* = 0.01).

### 3.2. Composition and Difference Analysis of Bacteria and Methanogens of In Vitro Experiment

**Figure 1:**
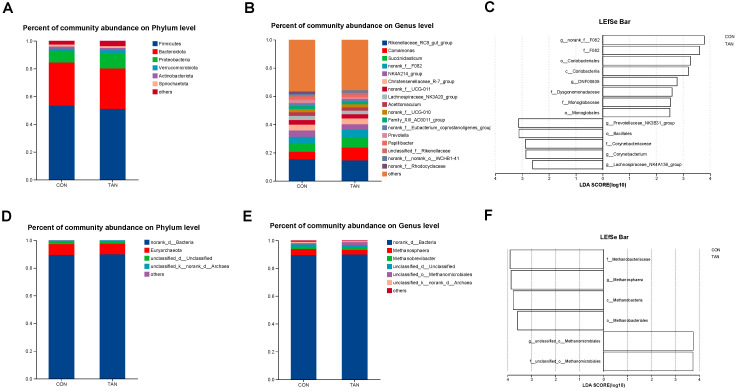
Effects of dietary tannic acid supplementation on the composition and diversity of rumen fermenting bacteria and methanogens of in vitro experiment. (**A**,**B**) Differences in bacteria at phylum and genus levels. (**D**,**E**) Differences in methanogens at phylum and genus levels. (**C**,**F**) The LDA effect size (LEfSe) analysis for bacteria and methanogens. *p* < 0.05 and LDA score > 2.50 were presented. CON, basic diet without TAN; TAN, substrates with 0.15% TAN (dry matter basis), *n* = 3.

In the in vitro fermentation experiment, Firmicutes and *Rikenellaceae_RC9_gut_group* were the bacteria with the highest relative abundance at the phylum level (Figure 1A) and genus level (Figure 1B), respectively. The methanogens with relatively high abundance at the phylum level (Figure 1D) and genus level (Figure 1E) were *Euryarchaeota* and *Methanosphaera*. LEfSe analysis of bacteria (Figure 1C) revealed a significant increase in the relative abundance of *Prevotellaceae*_*NK3B31*_*group* and *Lachnospiraceae_NK4A136_group* in CON compared with TAN (LDA > 2.50, *p* < 0.05). In contrast, the relative abundance of *F_082*, *Coriobacterlia*, and *Monoglobaceae* was significantly decreased (LDA > 2.50, *p* < 0.05). Additionally, LEfSe analysis of methanogens (Figure 1F) showed that *Methanosphaera* and *Methanobacteriaceae* had significantly higher relative abundance in CON than in TAN (LDA > 2.50, *p* < 0.05).

### 3.3. Growth Performance

Compared with CON, the ADG of the TAN group showed an increasing trend both in the d 0 to 30 (*p* = 0.06) and d 31 to 65 (*p* = 0.07) of the in vivo experiment. Moreover, throughout the experiment, the ADG of TAN group was significantly increased (*p* = 0.04) compared with CON (Table 3).

### 3.4. Slaughter Performance and Meat Quality

As shown in Table 4, compared to CON, the dressing percent (*p* = 0.03) and net meat percentage (*p* = 0.02) were significantly increased in TAN group, and the lightness of the longissimus dorsi muscle of the TAN group showed an increasing trend (*p* = 0.06) compared to CON, while the redness and yellowness showed no statistical difference between the two groups (*p* > 0.10).

### 3.5. Nutrient Digestibility

The results (Table 5) showed that there was no significant difference in the digestibility of DM, CP, NDF, ADF, OM, and EE between the TAN group and the CON group (*p* > 0.05).

### 3.6. Serum and Liver Antioxidant Capacity

There were no significant differences in the serum SOD, T-AOC, MDA, and BUN of beef cattle between the CON and TAN groups (*p* > 0.05). The MDA content in the liver of beef cattle in the TAN group significantly decreased (*p* = 0.02) compared with CON (Table 6).

### 3.7. Serum and Liver Immunity and Anti-Inflammation Capacity

As shown in Table 7, compared with CON, serum IL-10 showed a significantly lower trend (*p* = 0.08), while serum IGF-1 showed an increasing trend (*p* = 0.05) in TAN group.

### 3.8. Serum Biochemical Indicators

The serum Glu in the TAN group significantly increased (*p* < 0. 01), and the serum INS showed a decreasing trend (*p* = 0.10) compared with CON (Table 8).

### 3.9. Rumen Fermentation Parameters

There was no significantly different in the rumen fermentation parameters (*p* > 0.05) between CON and TAN groups. However, the concentration of rumen NH_3_-N (*p* = 0.10) and propionate (*p* = 0.08) in TAN group showed an increasing trend compared with CON (Table 9).

### 3.10. Bacterial Sequencing 

For both CON and TAN group ruminant fluid samples, sequencing analyses were conducted, resulting in a cumulative total of 437,900 optimized sequences with an average length of 419 bp (Table S2). After random subsampling based on the minimum sequence count per sample and subsequent alignment with the Silva database, a total of 1778 OTUs were identified, encompassing 18 phyla, 31 classes, 65 orders, 111 families, 236 genera, and 497 species.

### 3.11. Bacterial α-Diversity 

**Figure 2:**
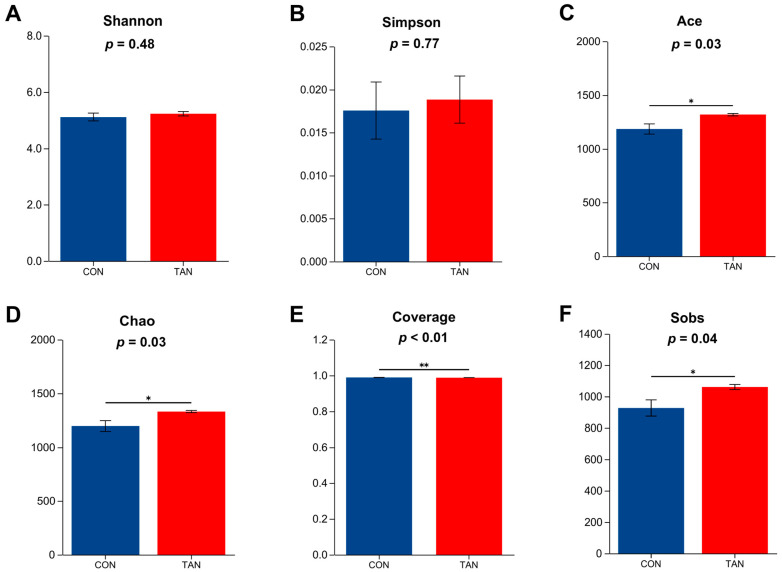
Effects of dietary tannic acid on rumen bacterial α-diversity in beef cattle. (**A**) Shannon index; (**B**) Simpson index; (**C**) Ace index; (**D**) Chao index; (**E**) Coverage index; (**F**) Sobs index. CON, basic diet without TAN; TAN, the basal diet supplemented with TAN at 0.20% DM. Sections marked with an asterisk (*) indicate *p* < 0.05, and (**) indicates *p* ≤ 0.01. *n* = 5.

As shown in Figure 2, compared with CON, the Ace, Chao, Coverage, and Sobs indexes were significant higher in TAN group (*p* < 0. 05), while the Shannon and Simpson indexes showed no significant difference between the two groups (*p* > 0.05).

### 3.12. Bacterial Composition and β-Diversity

**Figure 3:**
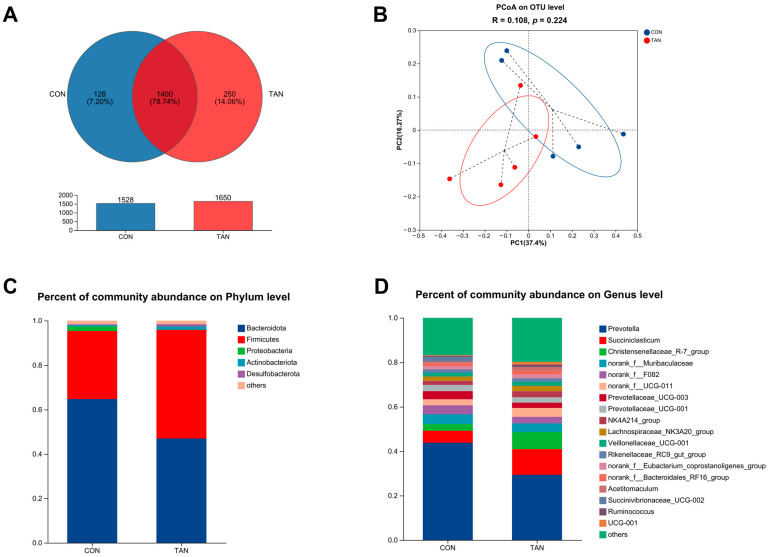
Effects of dietary tannic acid on the ruminal bacterial diversity and composition in beef cattle. (**A**) Venn analysis at the OTU level; (**B**) Principal Co-ordinate Analysis (PCoA) illustrating the distribution of OTUs; (**C**,**D**) taxonomic profiling at the phylum and genus levels. CON, basic diet without TAN; TAN, the basal diet supplemented with TAN at 0.20% DM. *n* = 5.

Venn diagrams identified 1400 shared OTUs, with CON and TAN presenting 128 and 250 unique OTUs, respectively (Figure 3A). Principal Co-ordinate Analysis (PCoA) at the OTU level revealed no significant differences between CON and TAN (PCoA: R = 0.108, *p* = 0.224) as shown in Figure 3B. Microbial distributions at the phylum (Figure 3C) and genus (Figure 3D) levels were visualized. In both CON and TAN samples, the top five microbial taxa at the phylum level comprised *Bacteroidota*, *Firmicutes*, *Proteobacteria*, *Actinobacteriota*, and *Desulfobacterota*. Meanwhile, at the genus level, the most prominent microbial groups in the CON and TAN samples included *Prevotella*, *Succiniclasticum*, *Christensenellaceae_R-7_group*, *norank_f_Muribaculaceae*, and *norank_f_F082*.

### 3.13. Bacterial Differential Analysis

**Figure 4:**
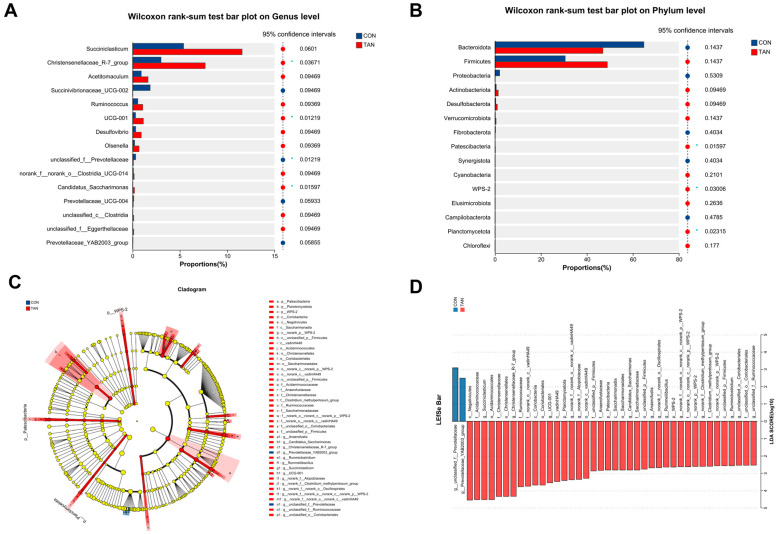
Effect of dietary tannic acid on rumen bacterial composition differences of beef cattle. (**A**,**B**) Divergences at the phylum and genus levels of microbiota. (**C**) Phylogenetic dendrogram; (**D**) Linear Discriminant Analysis Effect Size (LEfSe) evaluation. *p* < 0.05 and LDA score > 2.5 were presented. CON, basic diet without TAN; TAN, the basal diet supplemented with TAN at 0.20% DM; Bars marked with asterisks indicate statistical differences: * suggests *p* < 0.05, *n* = 5.

Compared to the TAN group, the CON group demonstrated a diminished abundance of *Patesclibacteria*, *WPS-2*, and *Planctomycetota* (*p* < 0.05) at the phylum level (Figure 4A). Concurrently, an elevation in the abundance of *Prevotellaceae* (*p* = 0.01) and a decrease in *Christensenellaceae_R-7_group, UCG-001*, and *Candidatus*_*Saccharimonas* (*p* < 0.05) were observed at the genus level (Figure 4B) in CON group. Furthermore, LEfSe analysis (Figure 4D) highlighted a significant augmentation in the abundance of *Prevotellaceae*_*YAB2003*_*group* in the CON group, with a notable decline in *Ruminococcaceae*, *Saccharimonadaceae*, and *Ruminiclostridium* (LDA > 2.50, *p* < 0.05). 

## 4. Discussion

An interesting observation of the in vitro experiment was that the TAN led to opposite changes in the rate and asymptotic gas production. TAN decreased the gas production rate but increased the cumulative gas production. Similar to our findings, Getachew et al. [28] found that adding TAN to alfalfa during a 72 h fermentation resulted in increased gas production and decreased rate, suggesting that rumen microbes could degrade TAN or be able to tolerate the effects of TAN. Conversely, Geerkens et al. [29] found that the inclusion of 167 mg/g gallic acid, a form of TAN, hindered in vitro rumen fermentation and suppressed gas production over a brief 24 h incubation period. Those results suggest that the impact of TAN on fermentation might be influenced by the length of fermentation, substrate, and the type of TAN used. For example, Deshpande and Salunkhe [30] reported that two types of TAN have different binding abilities to different types of starch. Our study also found that TAN tended to reduce methane production and suppressed the relative abundance of *Methanosphaera* and *Methanobacteriaceae* in the fermentation fluid. Numerous studies have demonstrated the methane-reducing effects of TAN in ruminants [31,32], which might be mainly due to the ability of TAN to inhibit methane production by binding to microbial cell proteins and enzymes, thereby inhibiting rumen methanogenic microorganisms.

Our study found that the addition of TAN in the diet improved the ADG of beef cattle. However, Tabke et al. [33] had different results; they reported that although TAN (30 or 60 g DM/steer daily, which was calculated to be approximately 0.30% or 0.60% of DM intake) numerically increased the ADG and carcass weight of beef cattle, the difference was not significant. Cattle from the current study were intact males, whereas Tabke et al. [33] fed castrated and implanted. Additionally, our experiment was relatively shorter (65 vs. 156 d) and started with a higher initial body weight (550 vs. 349 kg), which could also contribute to the differences between our study and Tabke et al. [33]. Interestingly, another study conducted by Barajas et al. [34] reported that the duration of TAN feeding also had different effects on the growth performance of beef cattle. They observed that feeding TAN for 100 d increased the ADG by 0.155 kg/d and resulted in a final carcass weight increase of 10.9 kg compared to the control group. However, when fed for 68 d, adding TAN in the diet led to a similar ADG but a reduction in DM intake by 0.62 kg/d, resulting in an increased feed conversion ratio by 0.015. Our results also found that the TAN group had an average increase of 10 kg in carcass weight per head and significantly improved dressing percent (+1.30%) and net meat percentage (+1.60%). The findings above indicate that the effects of dietary TAN on the growth and slaughter performance of beef cattle might be influenced by the duration and dosage of feeding and the growth stage of the cattle. Research has found that excessive dietary TAN could potentially reduce feed intake and even lead to toxicity in ruminants [35]. However, the lack of dry matter intake data in our research posed certain constraints in elucidating the effect of TAN on the ADG of fattening cattle. Future studies should consider incorporating dry matter intake to provide a more comprehensive evaluation of the impact of TAN on the ADG of beef cattle. In our research, the observed absence of adverse effects of TAN on growth and slaughter performance might be attributed to the conservative TAN dosage used and the fact that we fed cattle in the later stage of fattening with larger body weights.

Nutrient digestibility is a primary indicator for measuring the feed efficiency and growth performance of beef cattle [36]. Our research demonstrated that 0.20% of TAN in the diet did not affect the digestibility of CP and other nutrients of beef cattle, which was different from Yang et al. [5], who found that adding 0.65%, 1.3%, and 2.6% TAN in diet reduced CP digestibility of beef cattle, and 2.6% TAN even reduced DM and OM digestibility. Similarly, Zhou et al. [2] observed a decrease in DM, OM, and CP digestibility in beef cattle fed with 1.69% TAN. The decline in nutrient digestibility due to dietary TAN might be attributed to incomplete digestion and degradation of TAN-bound components, such as CP, minerals, and polysaccharides (cellulose, hemicellulose, pectin, starch) in the rumen [29,30]. However, a study conducted on sheep indicated that adding 1 and 2 g/kg of European chestnut extract (a hydrolysable form of TAN) to the diet had no effect on CP, OM, NDF, and ADF digestibility [37]. Overall, the impact of TAN on digestibility might be influenced by the dosage of supplementation and feeding conditions. Further research into the relationship between TAN and dietary components might reveal greater potential for understanding the effects of TAN on nutrient digestibility in beef cattle.

MDA, a product of lipid peroxidation, is frequently employed as a marker for oxidative stress in ruminants [38,39]. In this study, we found that diet TAN reduced the content of liver MDA in beef cattle, indicating that TAN improved the endogenous antioxidant status of beef cattle. Our finding was consistent with the results reported by Liu et al. [40], who found that adding chestnut tannins (10 g/kg, DM basis) in the diet reduced MDA concentrations in the plasma and liver of lactating cows, thereby improving the antioxidant status. Those results might be attributed to the ability of TAN to inhibit the formation of superoxide in the body and lipid peroxidation [41]. The chemical structure of tannin, including the O-dihydroxy structure in the B ring and the galloyl groups, might be involved in the activity of inhibiting lipid peroxidation, suggesting that they are important determinants for radical scavenging and antioxidative potential [42]. Previous studies have shown that supplementing TAN in the diet can enhance antioxidant capacity in the plasma or liver of dairy cows [43] and goats [44]. While past research has seldom reported on the ability of dietary TAN to mitigate lipid peroxidation in the liver of beef cattle, our study presents a novel finding in this regard. The potential of TAN to inhibit liver lipid peroxidation in beef cattle may be attributed to its gallic acid composition. This component can scavenge free radicals and bolster endogenous antioxidant defense mechanisms against them [43].

Blood glucose levels are influenced by transient shifts in insulin and glucagon concentrations, which react to the body’s nutrient requirements and availability [45,46]. In our study, beef cattle from the TAN group exhibited elevated blood glucose levels, which was constant with the finding of Reynolds et al. [47], indicating that diet TAN might influence the glucose metabolism of ruminants. Previous research has indicated that persimmon tannins can positively impact glucose metabolism in mice [48]. Additionally, hydrolyzed tannin extracts at a concentration of 1 μg/mL notably enhanced glucose transport in a pig intestinal cell model [49]. For ruminants, glucose is primarily sourced from propionate via gluconeogenesis in the liver [42]. This implies that TAN might boost the absorption and use of propionate generated in the rumen, subsequently enhancing hepatic gluconeogenesis. However, since our study lacked feed intake data and other metrics pertinent to glucose metabolism, this hypothesis warrants further investigation in future research. Dietary TAN could be metabolized by microorganisms in the rumen, releasing phenolic compounds such as gallic acid, pyrogallol, and resorcinol [50,51], and excessive degradation products of TAN might exceed the detoxification capacity of the liver [52], leading to symptoms of toxicity in ruminants [53]. It is worth noting that the TAN added in our experiment had no adverse effects on the immune, inflammatory, and biochemical parameters (BUN, TC, TG, TP, and ALB) in the serum and liver of beef cattle. This might be attributed to the relatively low feeding dose of TAN used in our experiment, approximately 0.036 g/kg body weight, which was much lower than the maximum recommended dose (<0.40 g/kg body weight daily) by Murdiati et al. [52].

The ruminal NH_3_-N had no significant change in our study, indicating that dietary TAN had less effect on rumen protein degradation. Similarly, Liu et al. [9] reported that adding 1.00% of chestnut tannin in the diet of Alcott sheep had no effect on ruminal NH_3_-N concentration, but a significant reduction was observed when the supplementation level was increased to 3.00%. This indicated that the protein-binding capacities of TAN might vary with different dosages or sources. Consistent with our findings, Aboagye et al. [54] reported that adding 0.25% and 1.50% chestnut tannin in the diet of beef cattle had no significant effect on the rumen T-VFA and the ratio of acetate to propionate. However, Pineiro-Vazquez et al. [4] reported that adding 1.00%, 2.00%, 3.00%, and 4.00% quebracho tannin in diet increased propionate and decreased the acetate-to-propionate ratio of beef cattle. Compared to the current study, Pieiro-Vazquez et al. [4] used heifers with an average weight of 295 kg, while our study used the beef cattle of the later finishing stage with an average weight of 550 kg. This difference in animal sex and growth stage might be the main reason for the observed discrepancies between the two studies. Moreover, our study indicates that incorporating TAN into the diet could potentially enhance the glucose metabolism capacity of beef cattle, which was substantiated by the elevated glucose concentrations observed in the serum of the TAN group. This heightened glucose metabolism might accelerate the absorption rate of propionate in the rumen [49], potentially explaining why there was not a significant uptick in the propionate concentration within the TAN group.

Higher Sobs, Chao, and Ace indices were observed in the TAN group, indicating that the addition of TAN in the diet increased the ruminal microbial diversity of beef cattle. Higher diversity of microbiota was generally associated with greater contributions to the health of the host [55], suggesting that the inclusion of TAN in the diet might help reduce the risk of diseases in beef cattle. *Prevotella* was one of the most abundant microbial genera in the rumen and played a crucial role in the digestion and utilization of substances such as starch, hemicellulose, pectin, and protein [56]. A study reported that feeding chestnut tannin-rich pine bark to sheep reduced the relative abundance of rumen *Prevotella* [57], which was also approved in the in vitro fermentation experiment [58]. TAN exerted strong inhibitory effects on the abundance of *Prevotella*, which might subsequently inhibit fiber degradation [59]. Consistent with previous research, we also observed a significant decrease in the relative abundance of *Prevotella* of the TAN group in both in vitro and in vivo experiments. *Succiniclasticum* played an important role in generating propionate from succinate [60], while *Ruminococcaceae* was considered highly specialized in degrading complex plant materials into VFA [61]. We observed an increase in the relative abundance of *Succiniclasticum* and *Ruminococcaceae* in response to TAN addition; however, the concentration of rumen VFA remained unaffected, which suggested that besides *Succiniclasticum* and *Ruminococcaceae*, other rumen bacteria might also play a crucial role in the formation of VFA. Nonetheless, dietary TA has been evidenced to instigate shifts in the rumen microbiota, exhibiting beneficial modulatory attributes [62]. The interaction of TAN with ruminal microbes, culminating in a reduction of methane and ammonia production and biohydrogenation of lipids in the rumen, stands pivotal in enhancing the growth performance of ruminants [1,63,64].

## 5. Conclusions

The inclusion of TAN in the diet increased in vitro gas production, improved the growth and slaughter performance of beef cattle, decrease liver MDA, and increased serum glucose concentration. Additionally, TAN suppressed the relative abundance of *Methanosphaera* and *Methanobacteriaceae* in vitro and enhanced ruminal microbial diversity and the relative abundance of *Ruminococcaceae* and *Succinivibrionaceae* in vivo. Our results suggest that dietary TAN might improve the performance and health status of beef cattle by mitigating liver lipid peroxidation, enhancing glucose metabolism, and promoting a balanced rumen microbiota for optimal fermentation. Further research could deeply explore the mechanism of TAN in promoting glucose metabolism and the anti-oxidation of beef cattle.

## Figures and Tables

**Table 1:** Diet composition and nutrition levels (%, DM basis).

Ingredient Composition	Content
Whole corn silage	30.0
Wheat bran	5.00
DDGS	5.00
Corn	28.0
Soybean meal	2.50
Corn germ meal	15.0
Beer residue	8.00
Cottonseed meal	5.00
Premix	0.20
NaCl	0.50
Magnesia	0.20
Limestone	0.60
Analyzed nutritional composition	
DM	51.2
CP	14.4
EE	2.89
NDF	38.0
ADF	20.0
Ca	0.54
P	0.28
Calculated nutritional composition	
NEg; Mcal/kg	1.62

DDGS, dried distillers grains with solubles; DM, dry matter; CP, crude protein; EE, ether extract; NDF, neutral detergent fiber; ADF, acid detergent fiber; Ca: calcium; P: phosphorus; The premix provided the following per kilogram of complete diet as Fe 12 g/kg, Mn 1 g/kg, Cu 1 g/kg, Zn 11 g/kg, I 30 mg/kg, Se 30 mg/kg, Co 20 mg/kg, Vitamin A 450,000 IU/kg, Vitamin D_3_ 60,000 IU/kg, Vitamin E 2000 mg/kg; NEg, Metabolic energy.

**Table 2:** In vitro fermentation parameters.

Item	Treatments		SEM	*p*-Value
	CON	TAN		
Gas production dynamic, mL/0.20 g DM				
GP_12_	33.4	35.1	0.51	0.40
GP_24_	36.2 ^b^	44.6 ^a^	0.72	<0.01
GP_48_	45.2 ^b^	51.6 ^a^	0.75	<0.01
GP_72_	47.2 ^b^	53.2 ^a^	0.81	<0.01
GP_96_	48.6 ^b^	54.1 ^a^	0.84	0.01
B	45.6 ^b^	52.3 ^a^	0.83	<0.01
C, h^−1^	0.10 ^a^	0.09 ^b^	0.002	0.02
Gas composition after 96 h of fermentation, %				
O_2_ and N_2_	3.52	12.4	3.06	0.11
CH_4_	23.1	19.4	1.22	0.10
CO_2_	53.4	47.1	2.19	0.11

GP_12_, GP_24_, GP_48_, GP_72_, and GP_96_ represent cumulative gas production of 0.2 g DM substrate at incubation times of 12, 24, 48, 72, and 96 h; B, the asymptotic gas production per 0.20 g DM substrate; C, the rate of gas production per hour; CON, basic diet without TAN; TAN, substrates with 0.15% TAN (dry matter basis). ^a,b^ Values with various superscripts in a row were significant differences (*p* < 0.05). *n* = 3.

**Table 3:** Effect of dietary tannic acid on the average daily gain of beef cattle.

Item	Treatments		SEM	*p*-Value
	CON	TAN		
BW, kg				
D 0	550	551	9.18	0.89
D 30	586	592	9.45	0.63
D 65	630	641	9.80	0.43
ADG, kg/d				
D 0 to 30	1.16	1.32	0.05	0.06
D 31 to 65	1.30	1.44	0.05	0.07
D 0 to 65	1.23 ^b^	1.38 ^a^	0.05	0.04

SEM, standard error of the mean; BW, body weight; ADG, average daily gain; CON, basic diet without TAN, *n* = 10; TAN, the basal diet supplemented with TAN at 0.20% DM, *n* = 9. ^a,b^ Values with various superscripts in a row were significant differences (*p* < 0.05).

**Table 4:** Effect of dietary tannic acid on slaughter performance and meat quality of beef cattle.

Item	Treatments		SEM	*p*-Value
	CON	TAN		
Slaughter performance				
BW before slaughter, kg	635	643	9.98	0.89
Hot carcass weight, kg	357	370	6.29	0.29
Net meat weight, kg	287	301	5.49	0.18
Dressing percent, %	56.2 ^b^	57.5 ^a^	0.37	0.03
Carcass meat rate, %	80.5	81.4	0.45	0.20
Net meat percentage, %	45.2 ^b^	46.8 ^a^	0.37	0.02
Meat quality				
Lion-eye area, cm^2^	154	156	1.91	0.48
pH_45min_	6.74	6.77	0.03	0.59
L*	33.1	35.2	0.67	0.06
a*	10.6	10.9	0.20	0.39
b*	7.18	7.09	0.16	0.71
Drip loss, %	13.9	13.4	0.29	0.24

SEM, standard error of the mean; L*, lightness; a*, redness; b*, yellowness. CON, basic diet without TAN; TAN, the basal diet supplemented with TAN at 0.20% DM; ^a,b^ Values with various superscripts in a row were significant differences (*p* < 0.05). *n* = 5.

**Table 5:** Effect of dietary tannic acid on nutrient utilization of beef cattle (%).

Item	Treatments		SEM	*p*-Value
	CON	TAN		
DM	52.98	54.04	2.07	0.73
CP	52.11	47.38	2.38	0.20
NDF	62.87	61.72	1.94	0.69
ADF	65.17	65.39	1.91	0.94
OM	49.56	50.20	1.90	0.82
EE	52.75	57.55	4.27	0.45

SEM, standard error of the mean; DM: dry matter; CP: crude protein; NDF: neutral detergent fiber; ADF: acid detergent fiber; OM, organic matter; EE: ether extract. CON, basic diet without TAN; TAN, the basal diet supplemented with TAN at 0.20% DM. *n* = 5.

**Table 6:** Effect of dietary tannic acid on serum and liver antioxidant capacity of beef cattle.

Item	Treatments		SEM	*p*-Value
	CON	TAN		
Serum				
SOD, U/mL	139	138	6.41	0.89
T-AOC, mmol/L	0.51	0.56	0.05	0.49
MDA, nmol/mL	3.64	3.82	0.32	0.71
Liver				
SOD, U/ mg of protein	225	237	8.25	0.14
T-AOC, mmol/ g of protein	0.73	0.66	0.04	0.23
MDA, nmol/mg of protein	1.37 ^a^	1.09 ^b^	0.14	0.02

SEM, standard error of the mean; SOD, superoxide dismutase; MDA, malondialdehyde; CON, basic diet without TAN; TAN, the basal diet supplemented with TAN at 0.20% DM; ^a,b^ Values with various superscripts in a row were significant differences (*p* < 0.05). *n* = 5.

**Table 7:** Effect of dietary tannic acid on serum and liver immunity and anti-inflammation indicators of beef cattle.

Item	Treatments		SEM	*p*-Value
	CON	TAN		
Serum				
IgA, g/L	1.21	1.19	0.10	0.96
IgG, g/L	7.66	9.77	0.65	0.11
TNF-α, pg/ml	80.11	72.26	9.35	0.72
IL-10, pg/ml	23.46	17.18	2.17	0.08
HSP-70, ng/ml	11.50	12.11	0.70	0.38
IGF-1, ng/ml	233.0	250.6	5.47	0.05
Liver				
IgA, mg/g	0.11	0.11	0.01	0.89
IgG, mg/g	0.75	0.68	0.04	0.34
TNF-α, pg/mg	78.34	65.48	5.66	0.17
IL-10, pg/mg	34.47	30.71	2.19	0.40
HSP-70, ng/mg	12.56	12.63	0.34	0.72
IGF-1, pg/mg	17.16	16.00	1.43	0.60

SEM, standard error of the mean; IgA, immunoglobulin A; IgG, immunoglobulin G; TNF-α, tumor necrosis factor-α; IL-10, interleukin-10; HSP-70, heat shock protein 70; IGF-1, insulin-like growth factor-1. CON, basic diet without TAN; TAN, the basal diet supplemented with TAN at 0.20% DM. *n* = 5.

**Table 8:** Effect of dietary tannic acid on serum biochemical indicators of beef cattle.

Item	Treatments		SEM	*p*-Value
	CON	TAN		
Glu, mmol/L	6.38 ^b^	7.87 ^a^	0.30	<0.01
INS, μIU/mL	10.2	9.96	0.43	0.10
BUN, mmol/L	5.25	5.39	0.44	0.83
TC, mmol/L	5.09	3.95	0.45	0.97
TG, mmol/L	0.22	0.14	0.03	0.28
TP, g/L	79.8	77.8	3.94	0.97
ALB, g/L	33.1	27.0	1.68	0.28

SEM, standard error of the mean; Glu, glucose; INS, insulin; BUN, blood urea nitrogen; TC, total cholesterol; TG, triglycerides; TP, total protein; ALB, albumin. CON, basic diet without TAN; TAN, the basal diet supplemented with TAN at 0.20% DM; ^a,b^ Values with various superscripts in a row were significant differences (*p* < 0.05). n = 5.

**Table 9:** Effect of dietary tannic acid on rumen fermentation parameters of beef cattle.

Item	Treatments		SEM	*p*-Value
	CON	TAN		
NH_3_-N, mmol/L	26.21	37.10	3.63	0.10
Acetate, mmol/L	45.71	55.14	7.36	0.62
Propionate, mmol/L	5.88	6.43	0.15	0.08
Isobutyrate, mmol/L	1.19	1.20	0.08	0.95
Butyrate, mmol/L	2.07	1.33	0.34	0.37
Isovalerate, mmol/L	0.25	0.23	0.01	0.71
Valerate, mmol/L	0.76	0.93	0.10	0.47
Acetate: Propionate	7.78	8.57	1.16	0.83
T-VFA, mmol/L	55.86	65.26	7.78	0.64

SEM, standard error of the mean; NH_3_-N, ammonia nitrogen; T-VFA, total volatile fatty acid. CON, basic diet without TAN; TAN, the basal diet supplemented with TAN at 0.20% DM. *n* = 5.

## Data Availability

The original manuscript of this study is included in the article and further information is available upon reasonable request to the corresponding author.

## Supplementary Materials

The following supporting information can be downloaded at: https://www.mdpi.com/article/10.3390/antiox12091774/s1, Table S1: Composition and nutrient levels of the in vitro basal fermentation substrate (%, DM basis); Table S2: Sample sequencing information of in vivo experiment.

## References

[1] Aboagye I.A., Oba M., Koenig K.M., Zhao G.Y.Y., Beauchemin K.A. (2019). Use of gallic acid and hydrolyzable tannins to reduce methane emission and nitrogen excretion in beef cattle fed a diet containing alfalfa silage. J. Anim. Sci..

[2] Zhou K., Bao Y., Zhao G.Y. (2019). Effects of dietary crude protein and tannic acid on rumen fermentation, rumen microbiota and nutrient digestion in beef cattle. Arch. Anim. Nutr..

[3] Makkar H.P.S. (2003). Effects and fate of tannins in ruminant animals, adaptation to tannins, and strategies to overcome detrimental effects of feeding tannin-rich feeds. Small Rumin. Res..

[4] Pineiro-Vazquez A.T., Jimenez-Ferrer G., Alayon-Gamboa J.A., Chay-Canul A.J., Ayala-Burgos A.J., Aguilar-Perez C.F., Ku-Vera J.C. (2018). Effects of quebracho tannin extract on intake, digestibility, rumen fermentation, and methane production in crossbred heifers fed low-quality tropical grass. Trop Anim. Health Prod..

[5] Yang K., Wei C., Zhao G.Y., Xu Z.W., Lin S.X. (2017). Effects of dietary supplementing tannic acid in the ration of beef cattle on rumen fermentation, methane emission, microbial flora and nutrient digestibility. J. Anim. Physiol. N..

[6] Jayanegara A., Leiber F., Kreuzer M. (2012). Meta-analysis of the relationship between dietary tannin level and methane formation in ruminants from in vivo and in vitro experiments. J. Anim. Physiol. Anim. Nutr..

[7] Al-Jumaili W.S., Goh Y.M., Jafari S., Rajion M.A., Jahromi M.F., Ebrahimi M. (2017). An in Vitro Study on the Ability of Tannic Acid to Inhibit Methanogenesis and Biohydrogenation of C18 Pufa in the Rumen of Goats. Ann. Anim. Sci..

[8] Pennington J.A.T., Fisher R.A. (2009). Classification of fruits and vegetables. J. Food Compos. Anal..

[9] Liu H.W., Dong X.F., Tong J.M., Zhang Q. (2011). A comparative study of growth performance and antioxidant status of rabbits when fed with or without chestnut tannins under high ambient temperature. Anim. Feed Sci. Technol..

[10] Velasco V., Williams P. (2011). Improving Meat Quality through Natural Antioxidants. Chil. J. Agr. Res..

[11] Sehrawat A., Sultana S. (2007). Abrogation of thioacetamide-induced biochemical events of hepatic tumor promotion stage by tannic acid in Wistar rats. J. Environ. Pathol. Toxicol..

[12] Calis I.U., Cosan D.T., Saydam F., Kolac U.K., Soyocak A., Kurt H., Gunes H.V., Sahinturk V., Mutlu F.S., Koroglu Z.O. (2016). The Effects of Monosodium Glutamate and Tannic Acid on Adult Rats. Iran. Red Crescent Med. J..

[13] Wang Z., Zhao Y., Lan X.Y., He J.H., Wan F.C., Shen W.J., Tang S.X., Zhou C.S., Tan Z.L., Yang Y.M. (2022). Tannic acid supplementation in the diet of Holstein bulls: Impacts on production performance, physiological and immunological characteristics, and ruminal microbiota. Front. Nutr..

[14] He L., Yang J., Chen W., Zhou Z., Wu H., Meng Q. (2018). Growth performance, carcass trait, meat quality and oxidative stability of beef cattle offered alternative silages in a finishing ration. Animal.

[15] Geng C.Y., Feng X., Luan J.M., Ji S., Jin Y.H., Zhang M. (2022). Improved tenderness of beef from bulls supplemented with active dry yeast is related to matrix metalloproteinases and reduced oxidative stress. Animal.

[16] He T., Long S., Yi G., Wang X., Li J., Wu Z., Guo Y., Sun F., Liu J., Chen Z. (2023). Heating Drinking Water in Cold Season Improves Growth Performance via Enhancing Antioxidant Capacity and Rumen Fermentation Function of Beef Cattle. Antioxidants.

[17] Silva B.C., Godoi L.A., Supapong C., Bitsie B., Valadares S.C., Schoonmaker J.P. (2023). Effect of a molasses-based liquid supplement on gastrointestinal tract barrier function, inflammation, and performance of newly received feedlot cattle before and after a transport stress. J. Anim. Sci..

[18] Zhou K., Bao Y., Zhao G.Y. (2019). Effects of dietary crude protein and tannic acid on nitrogen excretion, urinary nitrogenous composition and urine nitrous oxide emissions in beef cattle. J. Anim. Physiol. Anim. Nutr..

[19] Menke K.H., Raab L., Salewski A., Steingass H., Fritz D., Schneider W. (1979). Estimation of the Digestibility and Metabolizable Energy Content of Ruminant Feedingstuffs from the Gas-Production When They Are Incubated with Rumen Liquor Invitro. J. Agric. Sci..

[20] Vansoest P.J., Robertson J.B., Lewis B.A. (1991). Methods for Dietary Fiber, Neutral Detergent Fiber, and Nonstarch Polysaccharides in Relation to Animal Nutrition. J. Dairy Sci..

[21] Vankeulen J., Young B.A. (1977). Evaluation of Acid-Insoluble Ash as a Natural Marker in Ruminant Digestibility Studies. J. Anim. Sci..

[22] Broderick G.A., Kang J.H. (1980). Automated Simultaneous Determination of Ammonia and Total Amino-Acids in Ruminal Fluid and Invitro Media. J. Dairy Sci..

[23] Chen S.F., Zhou Y.Q., Chen Y.R., Gu J. (2018). Fastp: An ultra-fast all-in-one FASTQ preprocessor. Bioinformatics.

[24] Magoc T., Salzberg S.L. (2011). FLASH: Fast length adjustment of short reads to improve genome assemblies. Bioinformatics.

[25] Edgar R.C. (2013). UPARSE: Highly accurate OTU sequences from microbial amplicon reads. Nat. Methods.

[26] Wang Q., Garrity G.M., Tiedje J.M., Cole J.R. (2007). Naive Bayesian classifier for rapid assignment of rRNA sequences into the new bacterial taxonomy. Appl. Environ. Microbiol..

[27] Ørskov E.R., McDonald I. (1979). The estimation of protein degradability in the rumen from incubation measurements weighted according to rate of passage. J. Agric. Sci..

[28] Getachew G., Pittroff W., Putnam D.H., Dandekar A., Goyal S., DePeters E.J. (2008). The influence of addition of gallic acid, tannic acid, or quebracho tannins to alfalfa hay on in vitro rumen fermentation and microbial protein synthesis. Anim. Feed Sci. Technol..

[29] Geerkens C.H., Schweiggert R.M., Steingass H., Boguhn J., Rodehutscord M., Carle R. (2013). Influence of apple and citrus pectins, processed mango peels, a phenolic mango peel extract, and gallic acid as potential feed supplements on in vitro total gas production and rumen methanogenesis. J. Agric. Food Chem..

[30] Deshpande S.S., Salunkhe D.K. (1982). Interactions of Tannic-Acid and Catechin with Legume Starches. J. Food Sci..

[31] Jayanegara A., Goel G., Makkar H.P.S., Becker K. (2015). Divergence between purified hydrolysable and condensed tannin effects on methane emission, rumen fermentation and microbial population in vitro. Anim. Feed Sci. Technol..

[32] Malik P.K., Trivedi S., Kolte A.P., Mohapatra A., Bhatta R., Rahman H. (2022). Effect of an anti-methanogenic supplement on enteric methane emission, fermentation, and whole rumen metagenome in sheep. Front. Microbiol..

[33] Tabke M.C., Sarturi J.O., Galyean M.L., Trojan S.J., Brooks J.C., Johnson B.J., Martin J., Baggerman J., Thompson A.J. (2017). Effects of tannic acid on growth performance, carcass characteristics, digestibility, nitrogen volatilization, and meat lipid oxidation of steers fed steam-flaked corn-based finishing diets. J. Anim. Sci..

[34] Barajas R., Cervantes B.J., Arechiga S.C., Espino M.A., Flores L.R., Camacho A., Romo J.A. (2011). Effect of length feeding additional tannins-extract on feedlot-performance of finishing-bulls. J. Anim. Sci..

[35] Krueger W.K., Gutierrez-Banuelos H., Carstens G.E., Min B.R., Pinchak W.E., Gomez R.R., Anderson R.C., Krueger N.A., Forbes T.D.A. (2010). Effects of dietary tannin source on performance, feed efficiency, ruminal fermentation, and carcass and non-carcass traits in steers fed a high-grain diet. Anim. Feed Sci. Technol..

[36] Grossi S., Rossi L., Dell’Anno M., Biffani S., Rossi C.A.S. (2021). Effects of Heated Drinking Water on the Growth Performance and Rumen Functionality of Fattening Charolaise Beef Cattle in Winter. Animals.

[37] Sliwinski B.J., Kreuzer M., Wettstein H.R., Machmuller A. (2002). Rumen fermentation and nitrogen balance of lambs fed diets containing plant extracts rich in tannins and saponins, and associated emissions of nitrogen and methane. Arch. Anim. Nutr..

[38] Zhao H.D., Tang X.Q., Wu M.L., Li Q., Yi X.H., Liu S.R., Jiang J.Y., Wang S.H., Sun X.Z. (2021). Transcriptome Characterization of Short Distance Transport Stress in Beef Cattle Blood. Front. Genet..

[39] Liu L., Zhang W.J., Yu H.J., Xu L.J., Qu M.R., Li Y.J. (2020). Improved antioxidant activity and rumen fermentation in beef cattle under heat stress by dietary supplementation with creatine pyruvate. Anim. Sci. J..

[40] Liu H.W., Zhou D.W., Li K. (2013). Effects of chestnut tannins on performance and antioxidative status of transition dairy cows. J. Dairy Sci..

[41] Lau D.W., King A.J. (2003). Pre- and post-mortem use of grape seed extract in dark poultry meat to inhibit development of thiobarbituric acid reactive substances. J. Agric. Food Chem..

[42] Yokozawa T., Cho F.J., Hara Y., Kitani K. (2000). Antioxidative activity of green tea treated with radical initiator 2,2′-azobis(2-amidinopropane) dihydrochloride. J. Agric. Food Chem..

[43] Santillo A., Ciliberti M.G., Ciampi F., Luciano G., Natalello A., Menci R., Caccamo M., Sevi A., Albenzio M. (2022). Feeding Tannins to Dairy Cows in Different Seasons Improves the Oxidative Status of Blood Plasma and the Antioxidant Capacity of Cheese. J. Dairy Sci..

[44] Zhong R.Z., Xiao W.J., Ren G.P., Zhou D.W., Tan C.Y., Tan Z.L., Han X.F., Tang S.X., Zhou C.S., Wang M. (2011). Dietary Tea Catechin Inclusion Changes Plasma Biochemical Parameters, Hormone Concentrations and Glutathione Redox Status in Goats. Asian Austral. J. Anim..

[45] Drackley J.K., Overton T.R., Douglas G.N. (2001). Adaptations of Glucose and Long-Chain Fatty Acid Metabolism in Liver of Dairy Cows during the Periparturient Period. J. Dairy Sci..

[46] Huntington G.B., Harmon D.L., Richards C.J. (2006). Sites, rates, and limits of starch digestion and glucose metabolism in growing cattle. J. Anim. Sci..

[47] Reynolds D., Min B.R., Gurung N., McElhenney W., Lee J.H., Solaiman S., Bolden-Tiller O. (2020). Influence of tannin-rich pine bark supplementation in the grain mixes for meat goats: Growth performance, blood metabolites, and carcass characteristics. Anim. Nutr..

[48] Nishida S., Katsumi N., Matsumoto K. (2021). Prevention of the rise in plasma cholesterol and glucose levels by kaki-tannin and characterization of its bile acid binding capacity. J. Sci. Food Agric..

[49] Brus M., Frangez R., Gorenjak M., Kotnik P., Knez Z., Skorjanc D. (2021). Effect of Hydrolyzable Tannins on Glucose-Transporter Expression and Their Bioavailability in Pig Small-Intestinal 3D Cell Model. Molecules.

[50] Murdiati T.B., Mcsweeney C.S., Lowry J.B. (1992). Metabolism in Sheep of Gallic Acid, Tannic-Acid and Hydrolyzable Tannin from Terminalia-Oblongata. Aust. J. Agric. Res..

[51] Fay J.C., Alonso-Del-Real J., Miller J.H., Querol A. (2023). Divergence in the Saccharomyces species’ heat shock response is indicative of their thermal tolerance. bioRxiv.

[52] Murdiati T.B., Mcsweeney C.S., Campbell R.S.F., Stoltz D.S. (1990). Prevention of Hydrolyzable Tannin Toxicity in Goats Fed Clidemia-Hirta by Calcium Hydroxide Supplementation. J. Appl. Toxicol..

[53] Makkar H.P.S., Francis G., Becker K. (2007). Bioactivity of phytochemicals in some lesser-known plants and their effects and potential applications in livestock and aquaculture production systems. Animal.

[54] Aboagye I., Oba M., Castillo A., Koenig K., Beauchemin K. (2018). Effect of hydrolysable tannin with or without condensed tannin on animal performance and methane emission of beef cattle fed a high forage diet. J. Anim. Sci..

[55] McMullen C., Orsel K., Alexander T.W., van der Meer F., Plastow G., Timsit E. (2019). Comparison of the nasopharyngeal bacterial microbiota of beef calves raised without the use of antimicrobials between healthy calves and those diagnosed with bovine respiratory disease. Vet. Microbiol..

[56] Wu Q., Chen H., Zhang F., Wang W., Xiong F., Liu Y., Lv L., Li W., Bo Y., Yang H. (2022). Cysteamine Supplementation In Vitro Remarkably Promoted Rumen Fermentation Efficiency towards Propionate Production via *Prevotella* Enrichment and Enhancing Antioxidant Capacity. Antioxidants.

[57] Kong X., Zhang Y., Wang Z., Bao S., Feng Y., Wang J., Yu Z., Long F., Xiao Z., Hao Y. (2023). Two-step model of paleohexaploidy, ancestral genome reshuffling and plasticity of heat shock response in Asteraceae. Hortic. Res..

[58] Saminathan M., Sieo C.C., Gan H.M., Ravi S., Venkatachalam K., Abdullah N., Wong C.M.V.L., Ho Y.W. (2016). Modulatory effects of condensed tannin fractions of different molecular weights from a *Leucaena leucocephala* hybrid on the bovine rumen bacterial community in vitro. J. Sci. Food Agric..

[59] Dao T.-K., Do T.-H., Le N.-G., Nguyen H.-D., Nguyen T.-Q., Le T.-T.-H., Truong N.-H. (2021). Understanding the Role of *Prevotella* Genus in the Digestion of Lignocellulose and Other Substrates in Vietnamese Native Goats’ Rumen by Metagenomic Deep Sequencing. Animals.

[60] Xue Y.F., Lin L.M., Hu F., Zhu W.Y., Mao S.Y. (2020). Disruption of ruminal homeostasis by malnutrition involved in systemic ruminal microbiota-host interactions in a pregnant sheep model. Microbiome.

[61] Sanchez-Brinas A., Duran-Ruiz C., Astola A., Arroyo M.M., Raposo F.G., Valle A., Bolivar J. (2023). ZNF330/NOA36 interacts with HSPA1 and HSPA8 and modulates cell cycle and proliferation in response to heat shock in HEK293 cells. Biol. Direct.

[62] de Sant’ana A.S., Silva A.P.R., do Nascimento S.P.O., Moraes A.A., Nogueira J.F., Bezerra F.C.M., da Costa C.F., Gouveia J.J.D., Gouveia G.V., Rodrigues R.T.D. (2022). Tannin as a modulator of rumen microbial profile, apparent digestibility and ingestive behavior of lactating goats: A preliminary metagenomic view of goats adaptability to tannin. Res. Vet. Sci..

[63] Vasta V., Yanez-Ruiz D.R., Mele M., Serra A., Luciano G., Lanza M., Biondi L., Priolo A. (2010). Bacterial and Protozoal Communities and Fatty Acid Profile in the Rumen of Sheep Fed a Diet Containing Added Tannins. Appl. Environ. Microbiol..

[64] de Lucena A.R.F., Menezes D.R., de Carvalho D.T.Q., Matos J.C., Antonelli A.C., de Moraes S.A., Dias F.S., Queiroz M.A.A., Rodrigues R.T.S. (2018). Effect of commercial tannin and a pornunca (*Manihot* spp.) silage-based diet on the fatty acid profile of Saanen goats’ milk. Int. J. Dairy Technol..

