# Supplementary material for: Dietary Supplementation of Tannic Acid Promotes Performance of Beef Cattle via Alleviating Liver Lipid Peroxidation and Improving Glucose Metabolism and Rumen Fermentation
Source: Antioxidants (Basel). 2023 Sep 18;12(9):1774. doi: 10.3390/antiox12091774 (PMC10526014; doi:10.3390/antiox12091774)
Supplement: Supplementary file 1 [file antioxidants-12-01774-s001.zip › antioxidants-2561004-supplementary.pdf]

Table S1 Composition and nutrient levels of the in vitro basal fermentation substrate (%, DM basis).

Ingredient composition	Content
Corn	28.15
Soybean meal	8.64
Jujube powder	7.64
Whole corn silage	32.96
Corn stalk	17.04
Salt	1.13
Premix	2.22
Calcium hydrogen phosphate	1.10
Sodium bicarbonate	1.12
Nutritional composition	
DM	49.53
CP	12.24
NDF	42.51
ADF	22.25
EE	2.84
Ca	0.50
P	0.31

DM: dry matter; EE: ether extract; CP: crude protein; NDF: neutral detergent fiber; ADF: acid detergent fiber; Ca: calcium; P: phosphorus. Premix 1: Fe 12 g/kg, Mn 1 g/kg, Cu 1 g/kg, Zn 11 g/kg, I 30 mg/kg, Se 30 mg/kg, Co 20 mg/kg, Vitamin A 450,000 IU/kg, Vitamin D3 60,000 IU/kg, Vitamin E 2000 mg/kg.

Table S2. Sample sequencing information of in vivo experiment

Sample	Sequence number	Base number	Mean length	Min length	Max length
CON-1	44,509	18,697,405	420	234	489
CON-2	44,320	18,758,727	423	303	499
CON-3	45,642	19,119,461	419	205	469
CON-4	43,825	18,384,805	420	232	540
CON-5	44,598	18,717,890	420	235	510
TAN-1	41,652	17,522,899	421	234	523
TAN-2	40,067	16,785,390	419	220	436
TAN-3	45,653	19,142,599	419	252	487
TAN-4	41,598	17,403,513	418	230	493
TAN-5	46,036	19,084,614	415	232	492
Mean	43,790	18,361,730	419	238	494
